# Measuring a Journey without Goal: Meditation, Spirituality, and Physiology

**DOI:** 10.1155/2015/891671

**Published:** 2015-06-07

**Authors:** Heather Buttle

**Affiliations:** ^1^School of Psychology, Massey University, Private Bag 102904, North Shore Mail Centre, Auckland 0745, New Zealand; ^2^Mind and Life Institute, Amherst College, 271 South Pleasant Street, Amherst, MA 01002, USA

## Abstract

The secular practice of meditation is associated with a range of physiological and cognitive effects, including lower blood pressure, lower cortisol, cortical thickening, and activation of areas of the brain associated with attention and emotion regulation. However, in the context of spiritual practice, these benefits are secondary gains, as the primary aim is spiritual transformation. Despite obvious difficulties in trying to measure a *journey without goal*, spiritual aspects involved in the practice of meditation should also be addressed by experimental study. This review starts by considering meditation in the form of the relaxation response (a counterpart to the stress response), before contrasting mindfulness research that emphasizes the role of attention and alertness in meditation. This contrast demonstrates how reference to traditional spiritual texts (in this case Buddhist) can be used to guide research questions involving meditation. Further considerations are detailed, along with the proposal that research should triangulate spiritual textual sources, first person accounts (i.e., neurophenomenology), and physiological/cognitive measures in order to aid our understanding of meditation, not only in the secular context of health benefits, but also in the context of spiritual practice.

## 1. Introduction

Mind-body therapies have become popular in a number of Western countries, with some estimates reporting that almost 20% of the US adult population have practiced a form of mind-body therapy in the past twelve months [1]. Mindfulness meditation practices are one such type of mind-body practices. Successful outcome studies from mindfulness based interventions, such as mindfulness based stress reduction (MBSR) and mindfulness based cognitive therapy (MBCT) [2, 3], have led to research inquiries regarding the cognitive processes that underlie the physical and mental health benefits of mindfulness practices. Studies to date have included both behavioral and physiological data focusing on key cognitive aspects of mindfulness practice, such as attention, memory, and emotion regulation, and have included secular and spiritual forms of mindfulness/meditation [4–13]. Yet, the Eastern/Buddhist traditions that mindfulness practices have typically been drawn from have a spiritual objective that goes beyond the alleviation of acute or chronic health problems (the usual focus of secular mindfulness practices). This raises questions around the purpose and goal of studying meditation and how traditional spiritual textual sources may help guide future investigations.

Buddhist mindfulness/meditation is practiced in the context of a moral and philosophical system that aims to solve existential angst and problems of egotism through acceptance of change and reduction of attachment via recognition of* nonself* [14]. Emerging from the practice of mindfulness is an appreciation that the duality we usually experience (mind/body being one such dualistic distinction) does not exist in an absolute sense, as all phenomena are interconnected and everything is impermanent and subject to change [14].

In many traditions the meditation practitioner embarks on a paradoxical journey where spiritual transformation takes place without a definable or measurable goal. Trungpa discusses this issue as a journey without goal, stating that

*when we refer to a journey… On the one hand we are talking about nonbeing, no world, nonexistence; and on the other hand we are discussing the process of the path, how we could proceed along a path and exert ourselves… If we split hairs in that way, there is no truth anywhere, none whatsoever. Let it just be that way; let us have contradictions [15].*

Given the increased interest in investigating the benefits and processes involved in Buddhist and other Eastern traditions, it is timely to review and reflect on how measurement of this journey without goal can be achieved within an empirical framework.

## 2. The Relaxation Response

In the 1970s physiological research by Herbert Benson at Harvard Medical School led to the creation of a prominent and widespread meditation technique to elicit what he termed the relaxation response (RR), a counterpart to the stress response (SR). The technique itself is simple and straightforward, involving a few key steps that include sitting quietly and comfortably, closing the eyes, breathing through the nose with awareness of the breath for 10–20 minutes, and not judging the success of the practice [16]. Benson's research on the RR during the 1970s showed a pattern of physiological change that seemed to act in opposition to physical reactions seen in response to stress. These physiological changes included reduction in blood pressure, resting heart rate, and oxygen consumption [17]. For example, a study of office workers with normal blood pressure showed that practice of the RR technique led to lower blood pressure compared to a control group [12].

Although Benson's research initially stemmed from investigations involving spiritual practitioners of Maharishi Mahesh Yogi's Transcendental Meditation, the RR technique was designed to be practiced by the individual in either a secular or spiritual context. The importance of Benson's approach needs to be considered with regard to the scientific and cultural context of these early studies. There would have been great skepticism about the utility of meditation, and so establishing positive effects with physiological measures and rebranding the practice as a relaxation response would have made the study of meditation more palatable, thus opening the door to future research. In fact, Benson's research over the ensuing decades continued to investigate physiological changes due to the RR and included related practices that were likely to elicit the RR (such as advanced practitioners of Buddhism [5, 18]). As technological advances increased, Benson was able to study other physiological effects, as well as biochemical and genomic activity changes [10, 19–24].

Regarding biochemical changes most research had concentrated on the release of cortisol, with research on Buddhist monks and Transcendental Meditation practitioners showing reduced levels of cortisol (i.e., the release of cortisol is associated with stress) [25, 26]. Later it was suggested that nitric oxide (NO) could mediate the RR's physiological effects [24] given that it is a short-lived nitrogenous free radical known to mediate physiological processes, such as cardiovascular, immune, and nervous system function [27]. Indeed, research revealed that volumetric oxygen consumption (VO^2^), which is considered to be a measure of RR elicitation, was negatively related to NO, where the decrease in VO^2^ associated with the RR was accompanied by an acute increase in the presence of NO [20]. These researchers hypothesized that “NO changes play a role in the consistent pattern of blood pressure reduction that is seen during RR elicitation.”

Research on genomic changes reported that the RR led to specific gene expression changes in both short and long term practitioners, suggesting that such changes may relate to long term physiological effects [21]. More specifically, Bhasin et al. report that RR practice “…enhanced expression of genes associated with energy metabolism, mitochondrial function, insulin secretion, and telemore [*sic*] maintenance, and reduced expression of genes linked to inflammatory response and stress related pathways…” [19]. This research provides the first evidence for what the authors argue is the RR evoking downstream health benefits through the improvement of mitochondrial energy production/utilization, which promotes mitochondrial resiliency through upregulation of ATPase and insulin function.

Other research from Benson's lab focused on meditation using MRI and fMRI. This research indicates that meditation activates neural structures involved in attention and the control of the automatic nervous system (e.g., dorsolateral prefrontal and parietal cortex, hippocampus/parahippocampus, temporal lobe, pregenual anterior cingulate cortex, striatum, and pre- and postcentral gyri) and that brain regions, including those associated with attention, were thicker in meditation participants than matched controls. This was most pronounced in older participants, raising the possibility that meditation might offset age-related cortical thinning. Hence, there is structural evidence for experience-dependent cortical plasticity associated with meditation practice [10, 23].

In considering these findings together, Dusek and Benson have provided a conceptual model, which integrates physiological and molecular level changes in relation to the RR and SR [28]. The aim of the model is to help guide mind-body research, specifically with health outcomes in mind. While the RR research of Benson and colleagues may appear to have only a secular/health interest this is not to say that the importance of faith and spirituality has not been considered. Not only did Benson's early research start out by investigating physiological effects of transcendental meditation, but also later work looked at other religious meditation practices. For example, Buddhist monks were studied in relation to specific and advanced meditation practices, including Tum-mo yoga, which involves producing an inner heat, where increases in finger/toe temperature of up to 8.3°C were observed, while another study reported that resting metabolism could be raised by 61% and lowered by 64% [5, 18]. These studies provide further evidence that meditation practices do lead to measurable physiological changes, where previously there had only been anecdotal accounts.

It could be argued that studying these physiological changes in meditators is similar to studying effects on free divers (many of whom use relaxation or yoga techniques to prepare themselves). In the case of studying free divers, understanding oxygen changes in the blood can help us understand how free divers achieve their goal by holding the breath for such a length of time that they are able to dive so deeply [29]. But do physiological changes in meditators reveal anything about the spiritual goal of meditators?

More direct consideration of spirituality and faith came from Benson's observation that around 25% of people practicing the RR “feel more spiritual” and these same people had “fewer medical symptoms than those who reported no increase in spirituality” [30]. More specifically, Benson detailed three ways in which a person's religious convictions/philosophy of life enhanced effects of the RR: (a) increased adherence and enjoyment of the routine amongst those with an appropriate philosophic/religious focus, (b) affirmative beliefs that led to* remembered wellness*, which would revive top down nerve cell firing patterns in the brain associated with wellness, and (c) faith in an eternal or life transcending force (being a soothing belief) got the fullest out of remembered wellness and disconnected unhealthy logic [30].

The concept of remembered wellness is a way to harness the potential of the placebo effect, and the description of how a person's spiritual beliefs can enhance the RR mirrors what Benson and Friedman describe as being the three components that bring about the placebo effect [4]. These three are “(a) positive beliefs and expectations on the part of the patient; (b) positive beliefs and expectations on the part of the physician or health care professional; and (c) a good relationship between the two parties.” In the former case remembered wellness is a positive term for placebo, with the emphasis on faith rather than the physician.

Even with these considerations given to the role of spirituality, it can appear that positive physiological changes are the primary goal of the meditation practice, and this is often true for secular practice, especially in therapy. Yet for many practitioners of meditation these physiological effects are secondary gains, with the primary gain being spiritual transformation. However, without an operational definition of what spiritual transformation is, it is arguable whether it can be meaningfully measured. It might even be the case that the body in a state of near-perfect homeostasis equates with spiritual or existential satisfaction. Although the focus on health benefits in past and current research may be partly due to funding implications, where health research is more likely to be funded than spiritual research, it is too easy to dismiss the possibility of investigating the spiritual aspect in any greater depth.

Highlighting the importance of considering meditation practices alongside the spiritual traditions and textual sources that they are drawn from, Britton et al. point out that Buddhist texts describe a state of relaxed alertness (a balance between hyper- and hypoarousal), yet modern uses have placed emphasis “…on the relaxing effects of meditation without as much attention to the arousing or wake-promoting effects” [7]. However, there is now a growing body of work that has focused on cognitive aspects of alertness through considering the role of attention in mindfulness practices.

## 3. Mindfulness, Attention, and Wakefulness

Not all research has emphasized the relaxation aspect of meditation, with many studies investigating practices that fall under the umbrella term mindfulness. The term is common to Buddhist traditions, where it forms part of the eightfold noble path to* awakening*, and was adapted by Kabat-Zinn in his therapeutic program to mean “…paying attention in a particular way, on purpose, in the present moment, and nonjudgementally [*sic*]” [31]. Initially, his program was called stress reduction and relaxation but was changed to mindfulness based stress reduction (MBSR) in order to reflect the awakening aspect [32], and in both his definition and other researchers attempts to operationally define and model mindfulness, attention has been identified as having a key role [32–34].

Reflecting the above definition's emphasis on attention and present moment awareness/openness, Bishop et al. presented an operational definition of mindfulness with two key components: sustained attention and orientation towards present moment experiences [33]. There is further elaboration of the role of attention in Shapiro et al.'s discussion of the mechanisms of mindfulness. They discuss a cyclic process where attention, intention, and attitude are interwoven and occur simultaneously [34]. The inclusion of intention overlaps with Benson's emphasis on the potential to harness the placebo effect where outcomes of mindfulness practice have been shown to correlate with the original intentions of the practitioner (e.g., those with the intention of self-regulation, or self-exploration, or self-liberation achieve their stated aim) [4, 35].

A variety of techniques and methods have been used to investigate the role of attention in mindfulness meditation practices [4, 6, 7, 9–11, 13, 23, 35–46]. This includes behavioral data that supports the hypothesis that mindfulness practice leads to efficiencies in the dorsal attention system involved in voluntary top-down attentional selection, as well as indicating that retreat practice develops and encourages the emergence of receptive attention (i.e., improved alerting), which corresponds to the ventral attention system [40].

While having more of a focus on the physiological rather than the cognitive, Benson's research also includes evidence for the role of attention, with findings indicating that there are changes to structures of the brain involved in attention, including increased cortical thickness with meditation experience [10]. This finding was most pronounced for older adults and suggests that meditation might offset cortical thinning. Similarly, a study of Zen meditators found that there was no correlation between age and grey matter volume, nor age and attentional performance [42]. This was despite significant negative correlations being found for the control group. It was noted that this effect was most prominent in the putamen, which is implicated in attention. The authors suggest that “…the regular practice of meditation may have neuroprotective effects and reduce the cognitive decline associated with normal aging” [42].

These attentional effects may also have implications for emotional processing and affective control, where research has shown that meditation reduces habitual responding [13], and Lutz et al. suggest that focused attention training, as often employed in some styles of meditation, may be related with a reduction in emotionally reactive behavior [11]. However, effects of meditation experience are not all linear. For example, in one study that showed decreased activation of the amygdala (associated with emotion) with meditation experience, there was also an inverted U-shape curve regarding experience and attention related activation (i.e., monitoring, engaging, and orienting) [6]. The group of meditators who had an average of 19,000 hours of practice showed greater activation than nonmeditators. However, when a group with an average of 44,000 hours practice was compared with the less experienced group of meditators, the more experienced group showed less activation.

Other research also indicates there are nonlinear changes in activation with increased meditation experience [6, 7, 47–49], with Britton et al. arguing that brain areas involved in tonic alertness (vigilant attention) are more active in meditators (early stages of training) than nonmeditators, but expert meditators show less activation “as meditative expertise becomes more proficient and effortless in later stages” [7]. One explanation for this finding is that brain areas that were not previously coactivated come to have increased connectivity [7, 49–52].

These findings regarding attention and alertness are quite significant, especially if we contrast the relaxation response and clinical applications that seek to increase sleep duration/depth, with Buddhist texts and aims of enlightened awakening. Britton et al. point out that Buddhist meditators view the need for less sleep as a sign of progress and that sleep research has established a positive correlation between attention and wakefulness [53]. Moreover, there is only weak evidence that mindfulness meditation and MBSR promote sleep [54, 55], leading Britton et al. to argue that the Buddhist term awakening “…is not a metaphor, but rather an iterative process of neuroplastic modifications and increased efficiency that supports a new level of perceptual sensitivity and insight” [7]. It is interesting that even when mindfulness meditation is placed in a secular context, reference to spiritual traditions, such as Buddhism, can act to illuminate the practice and guide research investigations. Other issues relating to attention-based aspects of meditation could also benefit from similar considerations of spiritual and contextual practice.

A further example is the classification of meditation. A common categorization is to divide meditation into focused attention (FA) and open monitoring (OM) [11, 56]. Generally, beginners will be introduced and establish a practice using FA techniques, where there is a particular object to focus attention on (e.g., the breath), whereas OM meditation does not require an object of focus, but an aware and nonreactive acknowledgment of moment to moment awareness. In the case of FA there is an effortful commitment to sustain attention that helps to still the active mind. Once a student has established this in their practice they are then better able to practice OM. Lutz et al. describe a development in practice where “the ‘effortful' selection or ‘grasping' of an object as primary focus is generally replaced by the ‘effortless' sustaining of an awareness without explicit selection” [11]. Differences have been found between FA and OM practices; for example, OM practitioners have been shown to have superior responses to unexpected stimuli compared to FA practitioners, reflecting a more distributed attentional focus [46].

However, care needs to be taken in making sure this division of meditation styles is not taken out of context of actual practice. There are overlapping features to the two types of meditation, and often a practice will deliberately and systematically include both aspects. For example, in the Vajrayana tradition (associated with Tibetan Buddhism) many students engage in deity meditation. This involves visualization, mantra chanting, and OM. Typically, a practice will begin with the reciting of a text that describes the visualization that is to be focused on. This recitation arrives at a* completion* stage where the visualization is held as an object of focus, while a mantra is chanted. This involves sustained attention and fits the description of a FA meditation practice. However, the practice always moves on to a* dissolution* stage, where the visualization is dissolved, the mantra ceases, and the practitioner remains in a state of OM. A beginner may spend more time on the FA aspects compared to a more experienced practitioner, but both are practiced together to some degree. Hence, it is important to consider the context of the practice and to refer to traditional texts that might provide insights. Note that a somewhat similar division has been made by Tomasino et al. in their meta-analysis comparing Buddhist practices (defined similarly to FA) with Hinduist practices (defined similarly to OM), which showed anterior activations for the former and posterior activations for the latter [57].

Another complicating factor is that meditation practices, such as the one described above, potentially involve other important cognitive processes that have been neglected by researchers in comparison to the focus on attention. Buttle makes the point that meditations involving the simultaneous coordination of visual (imagery) and verbal (mantra/chanting) practices are likely to involve working memory systems and notes that Buddhist texts often refer to mindfulness in the context of memory (e.g., remembering to return to the object of focus when the mind has wandered), whereas the secular practice of mindfulness has focused on attention [8]. Even the type of imagery used in a meditation can yield differences, with deity meditation (visualizing ones body/mind as that of a deity), but not mandala meditation (mentally imagining the environment), nor rig-pa meditation (awareness without an object of focus), yielding improved performance on visual and spatial working memory tests [58]. Such findings support the view that instead of adhering to strict categories, such as FA and OM, meditation should be viewed on a spectrum, which allows for consideration of techniques that are self-transcending in the sense that they aim to change their own activity and experience [58–60].

Many practices are also designed to increase feelings of loving-kindness, compassion, and equanimity and are likely to involve affective processing. Understanding the spiritual aims behind the evolution of these practices can help guide research questions and may offer a way to assess spiritual aspects of the practice alongside material health benefits. Currently, the author's lab is investigating physiological changes in relation to emotional and moral content, in order to get an impression of how meditation practices may affect moral appraisal, with a view to considering the potential for societal benefit (as emphasized in Mahayana Buddhism), as well as individual benefit of the practices.

## 4. Meditation and the Spiritual Journey: Further Considerations

In considering the physiological study of meditation in the context of spirituality, it is important to reflect on the role given to meditation. Many religions use some form of contemplation. However, it is rare for meditation to be the only element of focus for the spiritual practitioner, as the practice is usually accompanied by both an ethical and philosophical system. This is true of Buddhist traditions from where these secular mindfulness programs have borrowed their techniques. Yet, in terms of historical practice, Sharf has argued that it is unclear how many Buddhists meditated (maybe less than supposed) and it was uncommon for people to write about their own meditational experience [61]. Regarding contemporary accounts, Bronkhorst highlights a number of Buddhist traditions where meditation seems to have a limited role [62]. Furthermore, some mindfulness researchers are starting to investigate negative experiences that arise from practicing meditation, with Britton, as quoted by Rocha, pointing out that while there is an abundance of positive results, “…no one has been asking if there are any potential difficulties or adverse effects, and whether there are some practices that may be better or worse-suited (for) some people over others [*sic*]” and this is despite historical accounts, from various traditions, providing negative descriptions of meditation experience [63]. In a qualitative study of experiences of committed meditators, Lomas et al. observed that around 25% of experiences were negative and could be classified into four main types of negative problem: that meditation (1) was difficult to learn and practice, (2) led to troubling thoughts/feelings that were hard to deal with, (3) exacerbated mental health issues, and (4) in a few cases was linked to psychotic episodes. However, these negatives were also seen as part of the journey, and as challenges they led to spiritual transformation [64].

Just as the investigation of the alert, attentional aspect of meditation has been prompted by the Buddhist concept of awakening, further textual understanding of spiritual traditions involving meditation may help in guiding research and offering new hypotheses and insights. This could include research on the ethical and philosophical aspects of these spiritual traditions. This may proffer additional refinements within secular and therapeutic techniques but would also help build understanding of benefits of meditation for those whose practice has spiritual transformation as the primary goal.

With regard to ethics, Monteiro et al. discuss traditional and contemporary mindfulness noting that “the often-fierce criticisms of MBIs (Mindfulness-based interventions) have focused on a single theme: the omission of immediately apparent ethics…” [65]. This is seen as a problem by some Buddhist's, where viewing mindfulness as bare attention with no ethical framework is seen to have the potential to lead to wrong mindfulness (e.g., someone could use the practice to calm and focus the mind in order to carry out a negative action, such as killing). Monteiro et al. point out that ethics are interpreted differently by various Buddhist traditions, so there is no absolute code of ethics and that even without explicit ethics the implicit ethics in MBSR may positively affect moral reasoning [65, 66]. However, there has been no empirical comparison of meditators who do and do not have an explicit code of ethics, and this would be worth investigating in future, especially as some meditation practices have loving-kindness and compassion as their focus (e.g., the practice of* metta* in Theravadin Buddhism and the practice of deity meditation in Vajrayana Buddhism).

Regarding philosophy, the Buddhist aim of practice is to recognize the compounded nature of all phenomena, so that the impermanence (of all things) in our lives (including our life) is accepted, leading to the cessation of suffering (*Dukkha*) [14]. Suffering in this sense is a state of unsatisfactoriness where we continually grasp at our sense of self, as if we are permanent and nonchanging, when experience will inevitably and painfully contradict that belief [14]. Buddhist traditions place somewhat different emphasis on the philosophy (*the view*); for example, some traditions focus on the idea of* nonself*, whereas others focus on all phenomena being empty of an inherent nature [67]. To complicate this further, a tradition may include a number of graded philosophies in order to move the practitioner towards the experience of how reality actually is (empty of inherent existence), with the aim of eventually realizing this without further need for the prop of a philosophical structure (eventuating in* enlightenment*) [67]. While such philosophies are debated within Buddhist scholastic centers, they are also used alongside meditation, where meditation provides a method for realizing the view. Caution is taken not to rush the introduction of the view and meditation, with teaching “emptiness to the untrained” being a violation of the Mahayana ethical system [68] (for a detailed account of these philosophies progressive stages in relation to meditation, see Rimpoche and Hookham [67]).

Buddhist texts have also described stages of meditation with terminology and interpretations differing between Pali and Sanskrit based traditions. Similarly, within traditions there are different types of classification; for example, there are the nine stages of placing the mind, which places emphasis on the calming aspect of meditation (*shamatha*), similar to FA, and the four stages of realization arising, which places emphasis on the insight and wisdom aspect (*vipashna*), similar to OM [69, 70]. With regard to the latter, these four stages can be further broken down with each stage having three substages [70]. The first stage (*one pointedness*) is where the meditator is learning to develop strong mental concentration; the second stage (*simplicity/no complication*) is where they are learning the true nature of phenomena; the third stage (*one taste*) is where there is experience of all phenomena having the same essence (empty of inherent existence); and the fourth stage (*no meditation*) is where there is realization of the experience and nothing remains to be done [69, 70]. While practitioners will typically progress through these stages gradually, Buddhist texts also recognize two other types of practitioner: one who at the first stage simultaneously achieves the other stages (rare but stable), and one who moves both forwards and backwards skipping stages (unstable) [70]. This indicates that while research often correlates meditation experience with hours/years of practice, it may not capture the individual variability of practitioners' progress.

Therefore, given that there are these existing classifications of meditation experience, it would be useful to submit them to experimental study with physiological measures. Moreover, as these practices involve a high degree of self-reflection on the part of the meditator, it would be beneficial to compare behavioral and physiological measures with first person accounts (see Buttle for an example of a self-reflective account [71]). Some work in cognitive- and neurophenomenology has already begun [58, 72–74]. This approach actively involves “…the participant in generating and describing specific and stable experiential or phenomenal categories” and can “…provide a way for experimenters to better control and identify the subjective aspects of attention and emotion regulatory processes” [11]. For example, Louchakova-Schwartz was able to contrast two types of Vajrayana meditation, where one (rigpa) is empty of cognitive contents, but not cognition, while the other (deity meditation) involved the visualization of complex and meaningful images [58].

It seems that there are many questions remaining to be explored in relation to both secular and spiritual/religious practices of meditation. Investigations informed by spiritual practices have already seen shifts in emphasis (e.g., from relaxation to wakefulness), and future research may well benefit from further consideration of spiritually and traditionally based textual sources. There is even further potential for investigating stages of meditation informed by textual scholarship, first-person descriptions, and cognitive/physiological measures. Ideally, such research would contribute to the building of a psychological theory as called for by Bronkhorst:

* This psychological theory should not just be shorthand for neurological processes, nor do we want mere folk psychology (or worse: psychobabble). Understanding brain processes is not sufficient to understand psychological processes, least of all when major transformations like those referred to in the early Buddhist texts are concerned. We need a psychological theory that is yet scientific in the strictest sense [62]. *

However, Bronkhorst also cautiously notes that “the neurological changes that correspond to this radical psychological transformation may well be relatively minor” [62]. This is a view that seems consistent with the Zen idea that the state that is sought is quite ordinary [75] and with the idea that there is a paradox of the journey without goal [15]. Moreover, it also fits with the inverted U-shape findings regarding attention and expertise, demonstrating that as meditators gain experience there is an increase in activation regarding attention, but in expert meditators no such increase is seen as meditation becomes effortless.

## 5. Conclusion

Secular meditation techniques have been based on spiritual practices, with research indicating that the spiritual sources of these techniques can also be useful in shaping research questions. Behavioral and physiological measures have given insight into the cognitive and physical effects of practice, further contributing to our understanding of mind-body interactions. However, there are a vast number of questions that remain to be answered, both in terms of secular and spiritual practices. One avenue that is worthy of further exploration is the triangulation of information drawn from textual sources, first-person accounts, and experimental measures. Measurement of spiritual transformation in this context may turn out to be its own journey without goal (even the Buddha refused to answer questions on whether the self and body were the same or different [76]). However, as technical tools for measuring behavioral and physiological changes become more sophisticated and more sensitive, we should seize opportunities that offer the potential to increase our empirical understanding, whether it is to improve secular therapies or to offer insights into spirituality and the cognition of religion.
